# Supplementary material for: Reduced Vessel Density in the Mid-Periphery and Peripapillary Area of the Superficial Capillary Plexus in Non-Proliferative Diabetic Retinopathy
Source: J Clin Med. 2022 Jan 21;11(3):532. doi: 10.3390/jcm11030532 (PMC8836591; doi:10.3390/jcm11030532)
Supplement: Supplementary file 1 [file jcm-11-00532-s001.zip › jcm-1525131-supplementary.pdf]

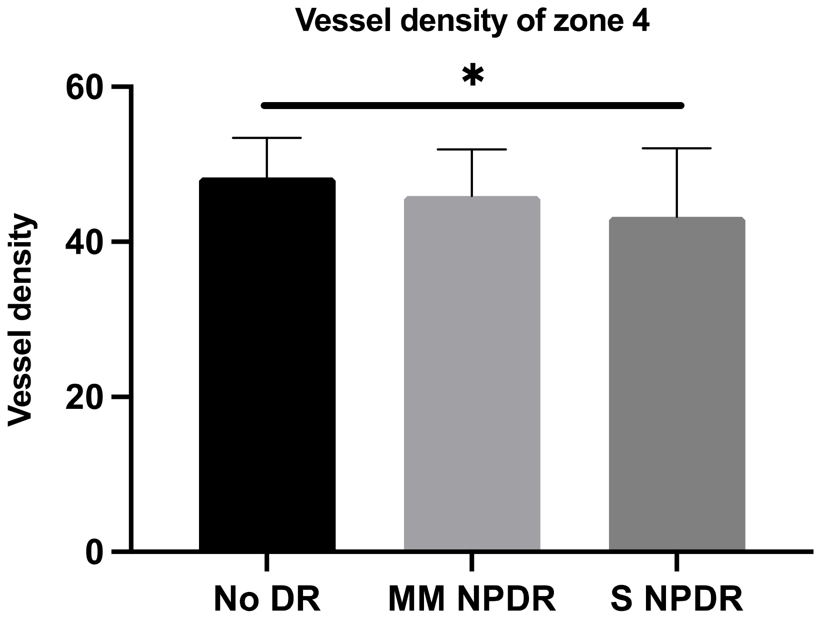

Figure S1. Mean vessel density with standard deviation for each group (without DR, mild and moderate NPDR, and severe NPDR) in zone 4, sub-analysis on 56 eyes of 56 patients (one eye included per patient). * $p < 0.05$. DR = diabetic retinopathy; MM NPDR = mild and moderate non proliferative diabetic retinopathy; S NPDR = severe non proliferative diabetic retinopathy.

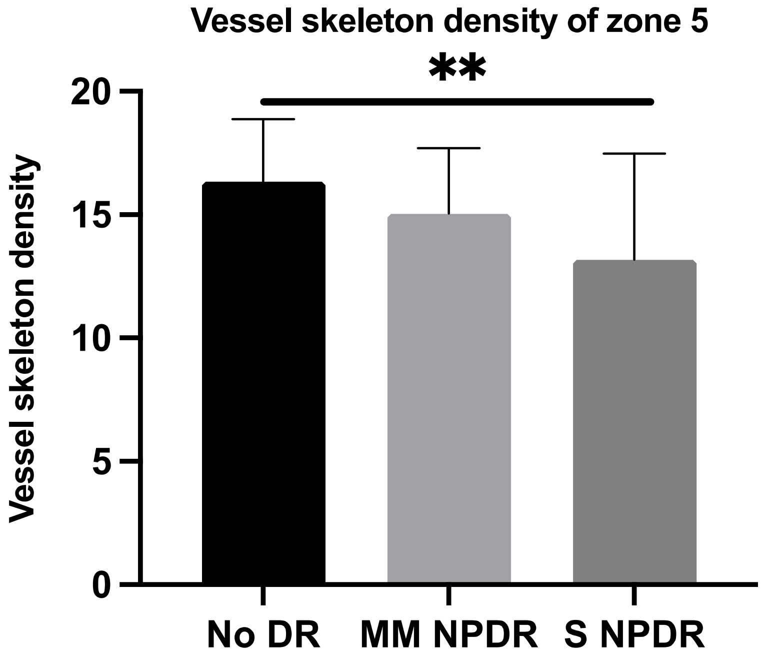

Figure S2. Mean vessel skeleton density with standard deviation for each group (without DR, mild and moderate NPDR, and severe NPDR) in zone 5, sub-analysis on 56 eyes of 56 patients (one eye included per patient). ** $p <0.01$. DR = diabetic retinopathy; MM NPDR = mild and moderate non proliferative diabetic retinopathy; S NPDR = severe non proliferative diabetic retinopathy.

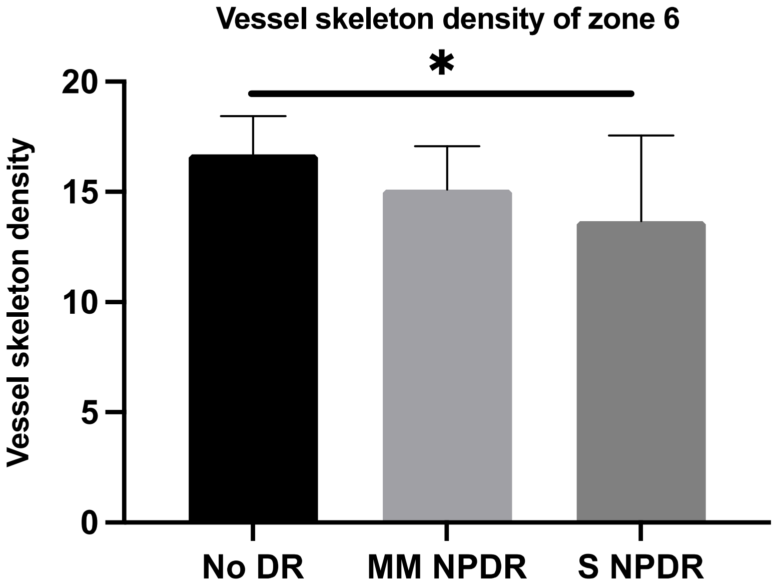

Figure S3. Mean vessel skeleton density with standard deviation for each group (without DR, mild and moderate NPDR, and severe NPDR) in zone 6, sub-analysis on 56 eyes of 56 patients (one eye included per patient). * $p < 0.05$. DR= diabetic retinopathy; MM NPDR = mild and moderate non proliferative diabetic retinopathy; S NPDR= severe non proliferative diabetic retinopathy.
